# Identification of rare X-linked neuroligin variants by massively parallel sequencing in males with autism spectrum disorder

**DOI:** 10.1186/2040-2392-3-8

**Published:** 2012-09-28

**Authors:** Karyn Meltz Steinberg, Dhanya Ramachandran, Viren C Patel, Amol C Shetty, David J Cutler, Michael E Zwick

**Affiliations:** 1 Department of Human Genetics, Emory University School of Medicine, Whitehead Biomedical Research Building, Suite 301, Atlanta, 30322, GA, USA; 2 Graduate Program in Population Biology, Ecology and Evolution, Emory University, 1510 Clifton Road, Atlanta, 30322, GA, USA; 3 Department of Genome Sciences, University of Washington, 3720 15th Avenue NE, Seattle, 98195, WA, USA

**Keywords:** Autism spectrum disorder, Massively parallel DNA sequencing, Rare variation, Evolutionary conservation

## Abstract

**Background:**

Autism spectrum disorder (ASD) is highly heritable, but the genetic risk factors for it remain largely unknown. Although structural variants with large effect sizes may explain up to 15% ASD, genome-wide association studies have failed to uncover common single nucleotide variants with large effects on phenotype. The focus within ASD genetics is now shifting to the examination of rare sequence variants of modest effect, which is most often achieved via exome selection and sequencing. This strategy has indeed identified some rare candidate variants; however, the approach does not capture the full spectrum of genetic variation that might contribute to the phenotype.

**Methods:**

We surveyed two loci with known rare variants that contribute to ASD, the X-linked neuroligin genes by performing massively parallel Illumina sequencing of the coding and noncoding regions from these genes in males from families with multiplex autism. We annotated all variant sites and functionally tested a subset to identify other rare mutations contributing to ASD susceptibility.

**Results:**

We found seven rare variants at evolutionary conserved sites in our study population. Functional analyses of the three 3’ UTR variants did not show statistically significant effects on the expression of *NLGN3* and *NLGN4X.* In addition, we identified two *NLGN3* intronic variants located within conserved transcription factor binding sites that could potentially affect gene regulation.

**Conclusions:**

These data demonstrate the power of massively parallel, targeted sequencing studies of affected individuals for identifying rare, potentially disease-contributing variation. However, they also point out the challenges and limitations of current methods of direct functional testing of rare variants and the difficulties of identifying alleles with modest effects.

## Background

The rapid development of better methods of targeted enrichment and genome sequencing has made it possible to detect a more complete spectrum of genetic variation [1-3]. These approaches hold out the hope of uncovering the genetic basis of polygenic complex human diseases, including autism (OMIM 209850), a childhood-onset disorder characterized by impaired social interactions, abnormal verbal communication, restricted interests, and repetitive behaviors. Autism has an estimated prevalence of one percent [4,5], and one of its most striking epidemiological features is a four-fold excess of affected male individuals.

Autism, or the broader autism spectrum disorder (ASD) phenotype, is an example of a highly heterogenous, multifactorial disorder with substantial heritability [6-13], (see reviews in [14,15]). Recent reports, in which X-chromosome coding exons in individuals with ASD were sequenced, identified an excess of rare mutations predicted to be damaging in a variety of genes related to synaptic function [16,17]. To date, more than 100 different genes and genomic regions have been linked to this complex trait (see reviews in [18,19]). Despite these findings, however, most of the genetic risk for ASD remains unexplained. Recent studies of ASD genetics generally adopt one of three study designs. The first employs genome-wide association studies, which have identified a few loci of interest, but largely failed to replicate findings between studies [7,20,21]; a meta-analysis of these studies, with a total of over 2500 study subjects, reveals it is extremely unlikely that there is any common variant influencing autism susceptibility, with an odds ratio of greater than 1.5 [22]. The second design focuses on large but very rare (frequency usually less than one in a thousand in the general population) *de novo* and inherited copy number variants (CNVs). Numerous studies have now shown convincingly that this class of rare variation makes a significant contribution to autism susceptibility [23-34], explaining up to 15% of all ASD cases. Unfortunately, these studies also point to a highly heterogenous allelic architecture, as no single risk variant is found in more than 1% of surveyed cases. The third has applied exome sequencing to identify *de novo* or inherited variants that contribute to ASD [35-37]. Overall, although genetic studies have uncovered many candidate loci, much ASD heritability remains unexplained [15].

Neuroligin pathway genes, including the neuroligins, neurexins, and *SHANK* genes, are critical to synapse development and function [38-40]. Several rare mutations in neuroligin genes, including single nucleotide variants (SNVs), insertions, splice variants, and deletions of whole exons, have been implicated in the pathogenesis of ASD [41-49]. These mutations often segregate with ASD in families [41,42]; however, they are also associated with variable cognitive phenotypes, including intellectual disability (ID) [42,50], Tourette syndrome [46], and language disability [43]. Neuroligin’s binding partners, the neurexins, are also critical to synaptic function, and point mutations and copy number variants in neurexin genes have been linked to ASD [27,51-54]. In addition, variants in the *SHANK* genes that anchor the neuroligins in the post-synaptic density, are also implicated in ASD [29,55-58]. There is therefore substantial evidence that perturbations of genes from the neuroligin pathway contribute to ASD susceptibility.

Most prior studies of neuroligin pathway genes have focused primarily on either discovering variants located within exons or large CNVs that disrupt the locus (but see [59]). Here we sought to test the hypothesis that rare variants found at evolutionarily conserved sites within noncoding regions might act as ASD susceptibility alleles. Although the statistical power to test any single rare variant is low [60], direct functional testing and functional annotation of variant sites might reveal alleles with modest effects on ASD susceptibility. Based on the substantial male bias in autism prevalence and the fact that two of the suspected neuroligin pathway genes are on the X-chromosome, we performed comprehensive sequencing of the *NLGN3* (Xp13.1) and *NLGN4X* (Xp22.3) loci, including the coding exons, 5’UTR, 3’UTR, and flanking intronic and intergenic sequences in 144 male individuals with ASD obtained from the Autism Genetic Resource Exchange (AGRE) repository. Our motivation for this design arises from the fact that male individuals are hemizygous for the X-chromosome, while female individuals are heterozygous. Thus, recessive acting alleles would be expressed exclusively in male individuals and could therefore increase the prevalence of male ASD. The results of analysis of our sequence data identified a set of rare noncoding alleles at highly evolutionarily conserved sites that were worthy of further evaluation for their role in ASD susceptibility.

## Methods

### Selection of male individuals with ASD

We sequenced 144 male individuals from the AGRE multiplex collection [61]. Detailed diagnostic criteria can be found on the AGRE website [62]. Male individuals were chosen from families with two or more male affected sibpairs (ASPs) that either shared identical X-chromosome markers, DXS9895 and DXS9902, or shared > 98% of 52 genotyped single nucleotide polymorphisms (SNPs) in the Xp22.3 region. A total of 152 families fit these criteria. One individual was randomly chosen for sequencing if both affected siblings were equally affected; if they were not equally affected, those with autism, those classified as not quite autism (NQA), or those classified as broad-spectrum were chosen, in that order, to maintain consistency. Among the 152 samples, two were unavailable from the AGRE repository at the time of this experiment and six had global PCR failure. Thus we had a total of 144 samples for processing. All human samples used in our study were de-identified and obtained from the AGRE repository, which obtains consent to participate in research studies and publish findings. Our study was reviewed and approved by the Emory University Institutional Review Board (IRB) because it met the criteria for exemption under 45 CFR 46.101(b).

### Target DNA amplification for the *NLGN3* and *NLGN4X* loci

Long-range PCR (LPCR) primers for amplifying the target DNA sequence were designed using EmPrime [63]. All primers were obtained from Invitrogen (Carlsbad, CA, USA) list of all primers used in this experiment can be found in Additional file 1. 500 ng of sample DNA was added to 1X LA Taq buffer (TaKaRa Bio Inc., Otsu Shigh, JP), 250 μM dNTP Mix (TaKaRa Bio Inc., Otsu Shigh, JP), 400 nM of both forward and reverse LPCR primers, and 0.1 U/μl of LA Taq (TaKaRa Bio Inc., Otsu Shigh, JP). If the amplicon had a high GC content, we used 1X GC Buffer (TaKaRa Bio Inc., Otsu Shigh, JP) in place of 1X LA Taq buffer. PCR was performed using the following parameters: initial denaturation at 94°C for 2 minutes, 29 cycles of 94°C for 10 seconds, 68°C for 1 minute per kilobase (of amplicon size), and a final extension time of 5 minutes.

Amplification was confirmed using 1% agarose 96-well E-Gels (Invitrogen, Carlsbad, CA, USA). We determined the concentration of each amplicon using PicoGreen dsDNA Quantitation Kits (Invitrogen, Carlsbad, CA, USA) and the Tecan Ultra Evolution plate reader. An equimolar concentration of each fragment was then pooled by sample for a total DNA concentration per sample of 10 ug. Pooled amplicons were then purified using the Invitrogen PureLink PCR Purification Kit with the HC buffer.

### Sample preparation for Illumina sequencing

Pooled, purified samples were sheared to approximately 300 bp using the Covaris E210, and fragmentation was confirmed by running Agilent Bioanalyzer DNA 7500 chips (Agilent Technologies, Santa Clara, CA, USA). We performed end repair using the NEBNext DNA Sample Prep Reagent Set 1 (New England BioLabs, Ipswich, MA, USA) with 0.4 mM dNTP mix, 5 ul of T4 DNA Polymerase, 1 ul of DNA Polymerase I (Klenow) fragment, 5 ul of T4 Polynucleotide Kinase, and 1X T4 DNA ligase buffer. The reactions were incubated in a thermal cycler for 30 minutes at 20°C. Following incubation, the reactions were purified using a QIAquick PCR Purification Kit (Qiagen, Valencia, CA, USA). To the purified, blunt, phosphorylated DNA fragments, we added 1X NEB Buffer 2, 1 mM dATP (NEB, Ipswich, MA, USA), and 3 ul of Klenow fragment (NEBNext Set 1). Following a 30-minute incubation at 37°C, reactions were purified using a QIAquick MinElute Kit (Qiagen, Valencia, CA, USA). To the DNA we added 1X Quick Ligation Buffer (NEBNext Set 1), 10 ul of Index PE Adapter Oligo Mix (from the Multiplexing Sample Preparation Kit; Illumina), and 5 ul of Quick T4 DNA Ligase. The reactions were incubated for 15 minutes at room temperature, and then purified using the QIAquick PCR Purification Kit (Qiagen, Valencia, CA, USA). This protocol uses a 10:1 molar Adapter:DNA ratio based on the starting concentration of DNA. We used the Size Select 2% E-Gels (Invitrogen, Carlsbad, CA, USA) to remove all unligated adapters and to accurately select the 300-bp band. The 300-bp band was successfully removed, and then selectively enriched using PCR to amplify the amount of DNA in the library and attach the 6-base index tag into the adapter. To 10 ul of DNA we added 1X Phusion PCR Master Mix (Finnzymes, Thermo Scientific, Lafayette, Co, USA; NEBNext Set 1), 1 ul each of PCR Primer lnPE 1.0 and PCR Primer lnPE 2.0, and 1 ul of PCR Primer Index (from Mulitplexing Sample Preparation Kit; Illumina). PCR parameters were as follows for 30 cycles: 98°C for 30 seconds, 98°C for 10 seconds, 65°C for 30 seconds, and 72°C for 30 seconds, with a final extension time of 5 minutes at 72°C. Following incubation, samples were purified using a QIAquick PCR Purification Kit (Qiagen, Valencia, CA, USA), and enrichment was confirmed using the Agilent BioAnalyzer 7500 DNA chip. Four pM of enriched DNA was used for cluster generation and paired-end sequencing on the Illumina Genome Analyzer II (IGA)

### Analysis of Illumina sequence data

Raw base-calling data generated by IGA were used as input for mapping and alignment. Paired-end reads were mapped and variants were called relative to a reference sequence using PEMapper (Cutler DJ *et al*., in revision). Briefly, the PEMapper is composed of four interconnected programs. The first program prepared a hashed index of the target sequence, the second program generated a list of potential mapping locations for each read. In the third stage, a Smith-Waterman alignment was performed at each potential location to determine the optimal position and alignment score. The output of the third stage, consisting of the pileup statistics of each base (number of reads where each nucleotide (A, C, G, T) was seen, together with the number of times that each base appeared deleted or an insertion immediately following the base) was used to make the genotype calls.

In total, 99.7% of target bases had at least 8X coverage, with a median depth of coverage of 452. SNVs and small insertions and deletions (indels) were annotated using the Sequence Annotator (SeqAnt) [64]. Functional annotation from hg18 included the genomic position, amino acid change, presence or absence in dbSNP132, and conservation scores (PhastCons, PhyloP) for each variant base. Additional filtering using dbSNP135 was carried out using the Feb. 2009, GRCh37(hg19) assembly from the UCSC Genome Browser [65]. The SNVs at highly conserved sites had coverages of 198 to 1,354, with the user base (non-reference allele) being called in > 92% in the sequence reads at the corresponding variant sites. A list of all SNVs and indels are contained in Additional files 2 and 3, respectively. As a comparison, we downloaded 3’UTR variants in *NLGN3* and *NLGN4X* from 1,094 individuals sequenced and deposited into the 1000 Genomes database [66]. A total of 49 3’UTR variants (38 SNVs, 11 indels) were identified in the *NLGN3* and *NLGN4X* genes (see Additional file 4).

We used popgen_fasta2.0.c code to perform population genetic analyses. This code calculated Watterson’s estimator of the population mutation rate (Θw per site) as well as a point estimate for Tajima’s D as previously described [67]. Variants at highly conserved sites were validated independently by Sanger sequencing (Agencourt Bioscience, MA). PCR primers for validation were designed using the Primer 3 [68]. Additionally, we sequenced the mothers and affected and unaffected male siblings with the validated UTR variants to verify the segregation pattern with autism (see Additional file 5). We also sequenced the mothers and two affected male siblings for two rare novel intronic variants that fell within transcription factor binding sites to verify the segregation pattern with autism (see Additional file 6).

### Control genotyping

Control samples used for genotyping were from male adults of European descent who had been screened to rule out psychiatric disorders, and were obtained from the National Institute of Mental Health (NIMH) Human Genetics Initiative [69]. Genotyping was performed by the iPLEX Gold Method (Sequenom, San Diego, CA, USA) per the manufacturer’s instructions, using primers designed with the Sequenom Assay design 3.1 software (see Additional file 7). A positive control was included in each plate to confirm the sensitivity of the assay.

### Functional testing of 3’UTR variants in a luciferase assay

Luciferase assays were performed to check whether the novel UTR variants we identified had altered gene expression relative to a construct containing the reference sequence. Full-length UTR sequences were amplified for three rare variants in *NLGN3* (70306922 (C > T), 70306764 (A > G), and 70306767 (C > G), and two rare variants in *NLGN4X* (5818136 (A > G), 5820149/50 (CT > −−)). The amplified sequences were cloned in to the multiple cloning site, downstream of the luciferase (*luc2+*) gene in the pmirGLO expression vector (Promega, Madison, WI, USA). A full-length 3’UTR sequence amplified from an unaffected normal control sample was cloned in to the same vector as the wild type. The *NLGN3* variant (70306764/67) served as the control for non-conserved UTR variant. The presence of the novel variant site was confirmed by Sanger sequencing.

Cell culture, transfection, and luciferase assays were performed on two different cell lines, mouse Neuro2a and human embryonic kidney 293 (HEK293), following the manufacturer’s instructions as reported previously with minor modifications described below [70]. In short, HEK293 cells and Neuro2a cells were cultured at 37°C with 5% CO_2_ in DMEM and RPMI 1640 (Cellgro Mediatech, Manassas, VA, USA) respectively, supplemented with 10% fetal bovine serum (Cellgro Mediatech, Manassas, VA, USA). Twenty-four hours before transfection, 0.2*10^6^ cells were plated in each well of 48-well cell culture dishes. Transfections were carried out using Lipofectamine™ 2000 in Opti-MEM (Invitrogen, Carlsbad, CA, USA) using 500 ng of plasmid. Just before each transfection, the old media were replaced with fresh media (DMEM or RPMI supplemented with 10% FBS). Twenty-four hours post transfection, cells were lysed with 250 ul of Passive Lysis Buffer (Promega, Madison, WI, USA), and cell debris were removed by centrifugation at 14,000 rpm for 5 minutes at 4°C. From each lysate, 20 μl of the cell extract were transferred into a luminometer tube, and 100 μl of Dual Luciferase Reporter Assay reagent (Promega, Madison, WI, USA) was added in each well. A manual luminometer (TD-20/20, Promega, Madison, WI, USA) was used to measure the luminescence over a 10-second period, with a delay time of 2 seconds. The luminometer reading was repeated after adding 100 ul of Stop and Glo reagent. For each lysate, the firefly luciferase activity was normalized to Renilla luciferase activity. We performed one independent transfection for each of the three 3’UTR alleles in two different cell lines (mouse Neuro2a and HEK293). Each transfection was replicated three times. A two-tailed, unequal variance Student’s *t*-test was performed to determine whether constructs with 3’UTR variants showed altered gene expression compared to constructs with the reference sequence.

### Functional annotation of intronic variants

Annotation of the variants was based on hg build 18 of the UCSC Genome Browser. Information regarding the Enhancer- and Promoter-Associated Histone Mark (H3K4me1 and H3K4me3) and the Transcription Factor Binding ChIP Seq were obtained from ENCODE Integrated Regulation tracks. Nuclease accessible site (NAS) information was obtained from the EIO/JCVI NAS Track, which annotates the location of NAS in the genome of human CD34+ and CD34- cells by NA-Seq technology. Conserved transcription factor binding sites (TFBS) were from human/mouse/rat (HMR) conserved TFBS track and were identified by searching within human-mouse-rat alignments using the position weight matrices (PWMs) from the TRANSFAC Matrix database (v7.0) [71]. The final z score can be interpreted as the number of standard deviations above the mean raw score for that binding matrix across the upstream regions of all RefSeq genes. The conserved transcription factor binding motif was displayed as a sequence logo [72] obtained at the Sequence Logo website [73].

## Results

**Figure 1:**
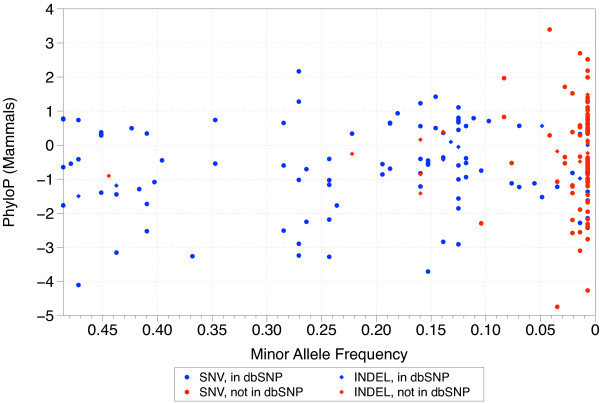
**Summary of single nucleotide variants (SNV) and insertions or deletions (indel) variation discovered at the*NLGN3*and*NLGN4X*loci in male individuals with autism spectrum disorder (ASD).** The frequency of SNVs and indels (minor alleles) in cases is plotted against their level of evolutionary conservation. Most common variation has already been discovered and exists in public databases (blue; circles and diamonds). Most of the rare variation at both loci was discovered in our study and not contained in public databases (red; circles and diamonds).

We sequenced the *NLGN*3 and *NLGN4X* loci in a sample of 144 male individuals with a diagnosis of autism; all the patient samples were obtained from the multiplex AGRE repository. We identified a total of 208 sites of variation, with 176 SNVs (see Additional file 2), and 32 indels (see Additional file 3). Overall levels of variation were estimated at 5.8 × 10^-4^ (Θ_w_ per site [74]), with an excess of rare variants as evidenced by a negative value for the Tajima’s D test statistic (−0.27) [75]. For the SNVs, a total of 37 (21%), had not been reported before (18 in *NLGN3* and 19 in *NLGN4X*). For the indels, a total of 22 (69%), had not been reported before (5 in NLGN3 and 17 in *NLGN4X*). As summarized in Figure 1, almost all common variation (> 5% frequency in our sample) is contained in dbSNP, whereas most rare variants (< 5%) have not been cataloged in dbSNP. 

Our study focused on previously undiscovered variation found at sites with elevated evolutionary conservation, so we did not follow up the 139 variants included in dbSNP. The only missense mutation we saw (*NLGN4X*, 5821532 G > A) had been reported before and, because of a nearby compensatory mutation, was not predicted to alter the primary structure of the protein [76]. Our data provide further evidence that coding sequence mutations at NLGN3 or NLGN4X that cause autism are very rare. Assuming the number of disease-causing coding mutations is Poisson distributed, we are 99% confident that the combined frequency of disease causing coding mutations at *NLGN3* and *NLGN4X* is less than 3% (no observations in 144 tries). Functional annotation of the remaining variant sites revealed that six SNVs and one indel were located at sites with elevated evolutionary conservation (PhastCons > 0.7, Table 1). 

**Table 1:** Summary of variants found at sites of increased evolutionary conservation

**Functional class**	**Position (hg18)**	**Variant type**	**PhastCons**	**PhyloP**	**Frequency in ASD cases**	**Frequency in NIMH control samples**
3’UTR	5818136	SNV	1	2.19	1/144	0/1440
3’UTR	5820149-50	Indel	1	0.85/1.76	1/144	ND
Intron	70284973	SNV	1	1.99	1/144	1/1416
Intron	70285256	SNV	0.98	0.90	1/144	0/1441
Intron	70288838	SNV	0.69	1.03	1/144	2/1441
Intron	70291656	SNV	1	2.52	1/144	0/1440
3’UTR	70306922	SNV	0.73	−0.47	1/144	0/1440

ASD, autism spectrum disorder; NIMH, National Institute of Mental Health; ND, Not Determined.

All of the rare variants were observed at a frequency of less than 1% in our ASD cases. To arrive at a better estimate of their population frequency, we genotyped six of the variants in a collection of 1,450 unaffected male controls obtained from the NIMH Human Genetics Initiative (Table 1). All of the variants genotyped had a frequency of less than 0.002. Thus, these data suggest that the variants we found are very rare in the general population.

### Functional analysis of 3’ UTR variants

Rare noncoding variants could act as autism susceptibility alleles by altering the level of expression of either *NLGN3* or *NLGN4X*. We sought to determine whether any of the three highly conserved 3’UTR variants of *NLGN3* (chrX:70306922) and *NLGN4X* (chrX:5818136, 5820149–50) could potentially lead to altered neuroligin expression in a luciferase reporter gene assay (Table 1). In addition to a construct containing the reference sequence, we also checked the expression of a construct containing two rare 3’ UTR *NLGN3* variants from a single individual (chrX:70306764/67) that were not located at evolutionary conserved sites, as an internal control. Each construct was tested in both mouse Neuro2a and human embryonic kidney 293 (HEK293) cells. The construct bearing the 3’UTR *NLGN3* variant (chrX:70306922) showed a trend for reduced luciferase activity the Neuro2a (*P* < 0.10) cells compared to the construct with the reference sequence (see Additional file 8). However, this result was not statistically significant and the average reduction (approximately 9%) was modest. Furthermore, the control construct showed a similar trend in the Neuro2a cells (*P* < 0.22). Neither construct showed a significant difference in the HEK293 cells (see Additional file 8). Inheritance of the 3’UTR *NLGN3* variants did not segregate with autism as shown in Additional file 5. None arose as *de novo* events in the ASD cases*.*

The 3’UTR *NLGN4X* variants did segregate with autism as shown in Additional file 5. None arose as *de novo* events in the ASD cases*.* A construct with the 3’UTR *NLGN4X* SNV (chrX:5818136) suggested a modest trend for increased luciferase activity in both the Neuro2a (*P* < 0.27) and HEK293 (*P*< 0.23) cells. However, the difference in expression was not statistically significant in either case (Additional file 8). The construct with the 3’UTR *NLGN4X* INDEL was not significant in either cell type (Additional file 8).

### Analysis of intronic variants

**Figure 2:**
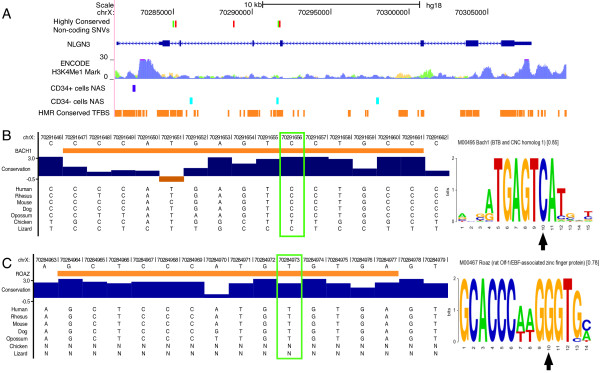
**Genomic location and evolutionary conservation of noncoding variants. (A)** The genomic position of highly conserved noncoding single nucleotide variants (SNVs) in *NLGN3* overlap with H3K4Me1 enhancer- and promotor-associated marks in various cell types (orange = H1 embryonic stem cells, green = human mammary epithelial cells (HMEC) and purple = human leukemia cells (K562)); CD34 cells nuclease accessibility sites (NAS) (purple = sites on CD34+ cells and blue = sites of CD34- cells); and human-mouse-rat (HMR) conserved transcription factor binding sites (TFBS) (orange blocks). The two variants that fall within HMR TFBS are highlighted in green. **(B)** Variant (chrX:70291656; also outlined in a green box) is located within a highly conserved 15-bp Bach1 TFBS (*z*-score 2.86, *P* < 0.003;). The sequence logo for the consensus Bach1 TFBS is shown to the right. The black arrow shows the position of the the variant (chrX:70291656) within this highly conserved binding motif. **(C)** Variant (chrX:70284973; outlined in a green box) is located within a highly conserved 14-bp Roaz TFBS (*z-*score 2.86, *P* < 0.003). The sequence logo for the consensus Roaz TFBS is shown to the right. The black arrow shows the position of the the variant (chrX:70284973) within this highly conserved binding motif.

We next sought to determine whether any of the four rare, intronic variants in *NLGN3* could act as autism susceptibility alleles. If so, we would predict that these variants should fall in regions identified as functional by the ENCODE Project [77]. All of the intronic variants are located within regions of enriched H3K4Me1 markers in H1 ES, HMEC, and K562 cells (Figure 2A). Regions with the mono-methylation of histone H3 lysine 4 are suggestive of enhancer and/or promotor activity due to the epigenetic modification of histone proteins [78]. 

One of the variants (chrX:70291656) was found to be located within the NAS of CD34- cells, and there are no common intronic variants located nearby. NAS are loci that are free of nucleosomes and are therefore hypothesized to allow *cis*-acting DNA to interact with trans-acting factors [79]. The same variant (chrX:70291656) also falls within a HMR conserved Bach1 TFBS (*z*-score 2.68, *P* < 0.004) (Figure 2B). Bach1 is a member of the BTB-basic leucine zipper transcription factor family and is a mammalian repressor of heme oxygenase 1 (HO-1) [80]. The intronic *NLGN3* variant chrX:70291656 found at the most highly evolutionary conserved site did segregate with autism as shown in Additional file 6.

An additional variant (chrX:70284973) falls within an HMR conserved Roaz TFBS (*z*-score 2.86, *P*< 0.003; Figure 2C). Roaz is a zinc finger protein that impairs the ability of the Olf-1/EBF transcription factor family to activate olfactory neuron-specific promotors [81]. This variant was found in one case and one control. There are no SNPs or repetitive elements in either of the regions of TFBS; the closest SNP annotated in dbSNP135 is located >150 bp upstream or downstream, and repetitive elements are >1 kbp from either variant. The intronic *NLGN3* variant chrX:70284973 also segregates with autism as shown in Additional file 6.

## Discussion

For the past 15 years, genomic studies of complex diseases have relied on a model in which common genetic variation contributes significantly to common diseases [82-84]. Based on this model, the systematic genotyping of common variants was perceived as the best way to begin characterizing the allelic architecture of complex human traits [85]. To make such experiments possible required the development of highly accurate, low-cost, high-throughput genotyping platforms and a catalog of common human genetic variation like the HapMap project [86,87]. Furthermore, because direct sequencing was not a viable strategy, assessing the role of common variation was really the only feasible genome-wide experiment. Thus, until recently the contribution of rare coding and noncoding variation to complex disorders like autism has gone largely unexplored.

While most quantitative traits, including human diseases, show substantial heritability in most populations, their allelic architecture remains poorly understood [88,89]. Haldane in the 1920s was the first to recognize that deleterious alleles of large effect will be maintained only at very low frequencies in the general population [90]. Copy number variation studies of ASD have identified variants with a large effect size, having odds ratios (ORs) often greater than 5.0 [23-34]. Much as Haldane would have predicted, these variants are quite rare, often occurring much less often than one in a thousand in the general population, a frequency generally consistent with a large effect locus at mutation selection balance.

At the same time, genome-wide association studies have shown that common variants with large effects are unlikely to exist in the human population for many disorders, although a large number of loci with alleles with much smaller ORs (< 1.2) remains plausible (see review in [91]). This is borne out in ASD, as genome-wide association studies have identified just a few loci of interest, which have largely failed to replicate findings between studies [7,20,21], whereas a meta-analysis suggests it is extremely unlikely that any common variant influences autism susceptibility with an OR of greater than 1.5 [15,22].

Here we used targeted, massively parallel sequencing of two X-linked genes, previously shown to harbor very rare point mutations causing ASD, to explore whether they might also have rare noncoding variants at evolutionary conserved sites that act as ASD susceptibility alleles. Using this approach we found a set of seven candidate variants, including three located in the 3’UTR, in the two genes examined among the 144 individuals sequenced (Table 1). As a comparison, a search for similar variants at highly conserved sites among 1,094 individuals sequenced and deposited into the 1,000 Genomes database identified a total of 49 3’UTR variants (38 SNVs, 11 indels) identified in *NLGN3* and *NLGN4X* genes (Additional file 4). None of the indels were found in highly conserved regions. A total of seven SNVs were found at highly conserved sites (PhastCons > 0.7), and two of the variants had an estimated minor allele frequency of 0.001. The remaining five variants did not have an estimated minor allele frequency. In considering this comparison, it is important to note that because we sequenced our samples to a far greater depth as compared to the 1,000 Genomes samples, our study had a greater probability of detecting rare variation.

Functional analysis of the 3’ UTR variants in a luciferase assay did not show a statistically significant difference in their expression (Additional file 8). The most likely interpretation is that these variants do not influence the risk of autism in these probands. However, two points are worth noting. First, under a quantitative genetic model of autism, we would not expect to find noncoding variants with large effects (that is, monogenic causes of autism), and instead might expect to find many alleles at many different loci, each with modest effects [15,92]. Second, our functional assays may be imperfect or insufficiently sensitive to reveal how these variants might act on their respective genes. Collectively these results point out the challenges of functional validation of alleles with modest effect sizes, even though the great heterogeneity of autism implies that such alleles should exist.

Our most promising intronic variant (chrX:70291656) is located in a highly conserved site in a TFBS that has been associated with neuronal dysfunction (Figure 2B). The Bach1 transcription factor protects cells from damage by activating HO-1. Bach1 dysregulation has been associated with Down syndrome (DS): Bach1 is significantly overexpressed in the fetal cortex of DS fetuses when compared to controls [93], whereas in another study, expression was significantly reduced in the frontal cortex of DS patients. In Bach1 knockout mice, expression of Bach1 mRNA was significantly higher in the olfactory bulb, but lower in the cortex versus wild-type mice, providing another link to olfaction [94]. It is possible the variant we found within the conserved TFBS influences olfactory neuron development and expression which could contribute to the sensory dysregulation phenotype of ASD. Interestingly, the affected individual harboring this variant in addition to being autistic, is intellectually disabled. He is diagnosed with sensory abnormalities including increased sensitivity towards acoustic and decreased sensitivity towards tactile senses. Still, our data do not demonstrate that this variant is functional through a direct experiment, but do predict that effects ought to be observed in such an experiment (for example, ChIP Seq).

Compared to children without neurodevelopmental disorders, children with ASD demonstrate olfactory and taste dysfunction [95,96]. Notably, in mice the *NLGN3* gene is expressed in all neurons of the olfactory bulb [40]. It is also interesting that we identified an intronic variant (chrX:70284973) that falls within a highly conserved TFBS related to olfactory neuron development (Figure 2C). Interestingly, this variant is predicted to increase binding efficiency at this TFBS. The Roaz transcription factor regulates both the temporal and spatial pattern of olfactory neuronal gene expression by binding to a consensus recognition sequence and modulating transcriptional activity [81,97]. Over 90% of children with ASD report sensory abnormalities, among them visual, auditory, tactile, and olfactory dysregulation (reviewed in [98]).

Our results highlight the importance of targeted sequencing of both coding and noncoding regions of candidate genes for complex, polygenic traits. Genetic studies of the X-chromosome have suggested that both rare and common X-linked variation may contribute to ASD [16,17,31,99-101], but much remains to be discovered. Although exome sequencing studies are now identifying point mutations, small indels, and *de novo* variants that contribute to ASD [35-37], these studies are limited by the regions they include in their exome capture chips, as well as biases in the capture efficiency of paralogous genes. Due to these constraints, these kinds of studies would have completely missed the noncoding variants we identified here. A study such as ours is also an important follow up for exome studies to assess the complete spectrum of genetic variation in genes known to harbor ASD-contributing mutations. These genes are often in candidate pathways related to neuronal development and function, and identifying mutations in noncoding and regulatory regions will likely shed more light on the etiology of ASD pathogenesis. As ASD is a polygenic trait, noncoding mutations probably play a role in the genetic contribution to ASD, in combination with other forms of genetic variation, including CNVs, coding mutations, and gene-disruptive indels that affect pathways related to brain development [102]. Still, our study points out that functional testing of rare variants remains challenging and not sufficiently high-throughput to perform this experiment on a genome-wide scale, especially when the effect sizes are modest. Finally, as whole-genome sequencing becomes increasingly cost effective and a more feasible experimental paradigm, detailed analyses of both coding and noncoding variation, as we have carried out here, can be expected to uncover ever more genetic variants that contribute to complex disorders like autism. These studies, however, will face significant challenges in direct functional testing of large numbers of these rare variants at highly conserved evolutionary sites.

## Conclusions

In conclusion, we used a highly targeted approach to identify rare variants that may contribute to ASD using massively parallel sequencing of the X-linked neuronal cell adhesion genes, *NLGN3* and *NLGN4X*. These data suggest that coding sequence variations in *NLGN3* and *NLGN4X* are rare. We identified three 3’UTR SNVs that did not show statistically significant effects in a luciferase assay. In addition, we uncovered intronic mutations that may affect regulatory regions, such as enhancer- and promotor-associated histone modification sites, NAS and TFBS. We suspect these variants may make modest contributions to ASD pathogenesis, as would be predicted by a quantitative genetic model of autism susceptibility. These data highlight one of the main challenges researchers face in the current era of next generation sequencing technology, namely establishing a direct link between the candidate variants identified and its contribution to the clinical phenotype of complex traits like autism.

## Abbreviations

AGRE: Autism Genetic Resource Exchange; ASD: Autism Spectrum Disorder; ASP: Affected SibPair; CNV: Copy Number Variant; DMEM: Dulbecco’s Modified Eagle’s Medium; DS: Down Syndrome; FBS: Fetal Bovine Serum; HEK: Human Embryonic Kidney; HMEC: Human Mammary Epithelial Cells; HMR: Human/Mouse/Rat; HO-1: Heme Oxygenase 1; ID: Intellectual Disability; indels: insertions and deletions; LPCR: Long-range Polymerase Chain Reaction; NAS: Nuclease Accessibility Site; NIMH: National Institute of Mental Health; NQA: Not Quite Autism; OR: Odds Ratio; PWMs: Position Weight Matrices; SNP: Single Nucleotide Polymorphism; SNV: Single Nucleotide Variant; TFBS: Transcription Factor Binding Site.

## Competing interests

The authors declare that they have no competing interests.

## Authors’ contributions

KMS, DR, and MZ participated in the design of the study. KMS performed the target DNA amplification and Illumina sequencing. KMS and DR performed validation of variant sites. DR performed genotyping and luciferase functional assays. Bioinformatic and statistical analyses were conducted by KMS, VCP, ACS, DJC, and MEZ. KMS, DR, DC, and MZ drafted the manuscript. All authors read and approved the final manuscript.

## Author’s information

Karyn Meltz Steinberg and Dhanya Ramachandran are co-first authors.

## Supplementary Material

Additional file 1 **Table showing long PCR primers.** Forward and reverse primers are listed for each of the regions sequenced. Click here for file

Additional file 2 **Table showing single nucleotide variants detected in 144 males with diagnosis of autism from the Autism Genetic Resource Exchange.** Contains variant position, dbSNP ID, functional annotation, and frequency in cases and controls. Click here for file

Additional file 3 **Table showing small insertion and deletion variants detected in 144 males with diagnosis of autism from the Autism Genetic Resource Exchange.** Contains variant position, dbSNP ID, functional annotation, and frequency in cases and controls. Click here for file

Additional file 4 **Table showing *NLGN3 *and *NLGN4X *3’UTR genetic variation from the 1000 Genomes database.** Contains variant position, dbSNP ID, PhastCons score, and estimated frequency. Click here for file

Additional file 5 **Figure showing segregation analysis of the highly conserved *NLGN3 *and *NLGN4X UTR *variants.** The segregation of the highly conserved UTR variants in *NLGN3* (chrX:70306922) and *NLGN4X* (chrX:5818136, chrX:5820149–50) with a diagnosis of autism were checked by sequencing the mother and affected and unaffected male siblings of the corresponding proband. The top base shown is the reference base, the bottom base shown is the variant base. Both the *NLGN4X* variants segregated with autism. We also tested segregation of the two control variants (chrX:70306764, chrX:70306767) with autism. DNA was not available for the unaffected sibling of the proband carrying the *NLGN4X* UTR variant (chrX:5820149–150). Click here for file

Additional file 6 **Figure showing segregation analysis of two highly conserved *NLGN3 *intronic variants.** The segregation of *NLGN3* intronic variants (chrX:70291656) and (chrX:70284973) with a diagnosis of autism. Both are located within transcription factor binding sites (TFBS). Click here for file

Additional file 7 **Table showing sequenom genotyping assay primers for single nucleotide variants discovered in *NLGN3 *and *NLGN4X *loci.** This table contains detailed information about each primer pair and extension primer. Click here for file

Additional file 8 **Figure showing the results of luciferase assays for one 3’UTR variant in *NLGN3 *and two 3’UTR variants in *NLGN4X.*** (A). Results of luciferase expression assay in Neuro2a cell lines. These constructs were tested against the reference allele constructs using a luciferase reporter assay. One of the *NLGN3* constructs (chrX:70306764/67) serves as a control for the variant at a non-conserved site. Error bars show 2 standard errors on either side of the mean value. (B). Results of luciferase expression assay in human embryonic kidney (HEK) cell lines. The experiment was similar to that described above. Click here for file
