# Supplementary material for: Identification of rare X-linked neuroligin variants by massively parallel sequencing in males with autism spectrum disorder
Source: Mol Autism. 2012 Sep 28;3:8. doi: 10.1186/2040-2392-3-8 (PMC3492087; doi:10.1186/2040-2392-3-8)
Supplement: Additional file 1 — Table showing long PCR primers. Forward and reverse primers are listed for each of the regions sequenced. [file 2040-2392-3-8-S1.rtf]

Supplementary Table 1. Long PCR primers (forward primers are even numbers, reverse primers are odd numbers)

ID	Primer Sequence	
NLGN3.0	5'-AAAGGTACCCAAAGTAGTGGTGAGCTAGGA-3'	
NLGN3.1	3'-GACAGAGGTGTGTATGGCAGGAGTTACTAAA-5'	
NLGN3.2	5'-CAACGAAGACTGTCTCTACCTGAACGTCTAT-3'	
NLGN3.3	3'-GAATGGAGTTACCTGGAGTGCTAGGAGAAT-5'	
NLGN3.4	5'-AGAGAGGAGGGAGGACTAAAAGAAGGACAG-3'	
NLGN3.5	3'-GATGATAGAAGGCGTAGAAGTAGGTAGGCG-5'	
NLGN3.6	5'-AGAGACTGTGTTCCTAGGTGACCATAGTGG-3'	
NLGN3.7	3'-CTGCCCATCTCCAGTGTACCATATTAGTGT-5'	
NLGN4.10	5'-GGAACCAGTGACCTCAAGAACTAGTCTGAA-3'	
NLGN4.11	3'-CTCACTGCTTAATAGATGAGGTAGCCACACAT-5'	
NLGN4.12	5'-CCTCATTTCTACTATGCGTACTCGCTGACT-3'	
NLGN4.13	3'-GAACTCCTAGCATACTCATTACGCTAAGGTGA-5'	
NLGN4.14	5'-GGAGCGCATTTCTACTTCTACCTTGAGTCTA-3'	
NLGN4.15	3'-GTCGGATCTAGTGGAGTCTGTAACTTACGTTG-5'	
NLGN4.16	5'-ATAGGGCATAGGTACTCAAGTGGGTAGGTG-3'	
NLGN4.17	3'-CTCAAGTAGCTCTCTGAGAGATCTCCATTCTG-5'	
NLGN4.18	5'-GACTTAGTATGTGAGACTGGAACTTCTCGGC-3'	
NLGN4.19	3'-CAGGAGCAGCGACTTATGTAGGGATAGTTA-5'	
NLGN4.20	5'-CTTCCAAACAACGGTGGTCTG-3'	
NLGN4.21	3'-CTGCCCGTCCACAGACTATTG-5'	
