# Supplementary material for: Identification of rare X-linked neuroligin variants by massively parallel sequencing in males with autism spectrum disorder
Source: Mol Autism. 2012 Sep 28;3:8. doi: 10.1186/2040-2392-3-8 (PMC3492087; doi:10.1186/2040-2392-3-8)
Supplement: Additional file 2 — Table showing single nucleotide variants detected in 144 males with diagnosis of autism from the Autism Genetic Resource Exchange. Contains variant position, dbSNP ID, functional annotation, and frequency in cases and controls. [file 2040-2392-3-8-S2.rtf]

Supplementary Table 2. Single nucleotide variants (SNVs) detected in 144 males with a diagnosis of autism from the Autism Genetic Resource Exchange (AGRE) 
Variant Position (hg18)	dbSNP ID (dbSNP 135)	Functional Class	PhastCons	Patient Frequency	Frequency in NIMH Control Samples	
5814756	rs5961881	Intergenic	0	65/144 (45.1%)	-	
5814904	rs112207600	Intergenic	0	3/144 (2.0%)	-	
5815085	rs73450207	Intergenic	0	23/144 (15.9%)	-	
5815164	   rs189863303	Intergenic	0	1/144 (0.69%)	-	
5815477	rs12115896	Intergenic	0	23/144 (15.9%)	-	
5815721	rs12860326	Intergenic	0	15/144 (10.4%)	-	
5816121	rs5916267	Intergenic	0	106/144 (73.6%)	-	
5816244	   rs143329759	Intergenic	0	15/144 (10.4%)	-	
5816610	rs7881510	Intergenic	0	1/144 (0.69%)	-	
5816615	rs5916268	Intergenic	0	103/144 (71.5%)	-	
5817073	rs5915617	Intergenic	0	109/144 (75.7%)	-	
5817317	rs5961378	Intergenic	0	109/144 (75.7%)	-	
5817768	rs5915618	Intergenic	0	17/144 (11.8%)	-	
5818136	-	3'UTR	1	1/144 (0.69%)	0/1440	
5818760	-	3'UTR	0	1/144 (0.69%)	-	
5818861	rs1882260	3'UTR	0	105/144 (72.9%)	-	
5818871	rs16983882	3'UTR	0	23/144 (15.9%)	-	
5819384	rs41309587	3'UTR	0	1/144 (0.69%)	-	
5819396	rs5961883	3'UTR	0	1/144 (0.69%)	-	
5819574	rs41305199	3'UTR	0	1/144 (0.69%)	-	
5820090	rs3810685	3'UTR	0.84	65/144 (45.1%)	-	
5820299	rs5916269	3'UTR	0	18/144 (12.5%)	-	
5820574	rs3810686	3'UTR	0	63/144 (43.7%)	-	
5820684	rs3810687	3'UTR	0.02	17/144 (11.8%)	-	
5820756	rs3810688	3'UTR	0	41/144 (28.4%)	-	
5821530	rs3747334	Silent	0	18/144 (12.5%)	-	
5821532	rs3747333	Replacement	0.52	18/144 (12.5%)	-	
5821807	rs61095217	Intron	0	23/144 (15.9%)	-	
5821965	rs5915619	Intron	0	18/144 (12.5%)	-	
5822641	rs6638575	Intron	0	106/144 (73.6%)	-	
5823297	rs56336632	Intron	0	23/144 (15.9%)	-	
5824098	rs5961884	Intron	0	109/144 (75.7%)	-	
5826043	-	Intron	0	2/144 (1.3%)	-	
5826140	rs6639537	Intron	0.09	109/144 (75.7%)	-	
5826328	rs6639538	Intron	0	87/144 (60.4%)	-	
5826553	rs67903143	Intron	0	50/144 (34.7%)	-	
5826634	-	Intron	0	1/144 (0.69%)	-	
5826639	     rs187767320	Intron	0	1/144 (0.69%)	-	
5827053	-	Intron	0	1/144 (0.69%)	-	
5827254	rs189328838	Intron	0	1/144 (0.69%)	-	
5827283	-	Intron	0	1/144 (0.69%)	-	
5827424	rs16983892	Intron	0	28/144 (19.4%)	-	
5827598	rs112498792	Intron	0	3/144 (2.0%)	-	
5828498	rs4603027	Intron	0	22/144 (15.2%)	-	
5828596	rs5915620	Intron	0	50/144 (34.7%)	-	
5828631	rs4451452	Intron	0.02	83/144 (57.6%)	-	
5828636	rs2369377	Intron	0	110/144 (76.3%)	-	
5828936	rs7878793	Intron	0	28/144 (19.4%)	-	
5829187	rs17315232	Intron	0	18/144 (12.5%)	-	
5829536	rs34078795	Intron	0	22/144 (15.2%)	-	
5829625	rs12390251	Intron	0	1/144 (0.69%)	-	
5829627	rs12394115	Intron	0	1/144 (0.69%)	-	
5830142	rs184727234	Intron	0.02	1/144 (0.69%)	-	
5830961	rs1921360	Intron	0	65/144 (45.1%)	-	
5830989	rs7888383	Intron	0	18/144 (12.5%)	-	
5831468	rs61741754	Silent	0.09	1/144 (0.69%)	-	
5831786	rs7049300	Silent	0.91	17/144 (11.8%)	-	
5832484	rs148155110	Intron	0	4/144 (2.7%)	-	
5832672	rs57049927	Intron	0	20/144 (13.8%)	-	
5832831	rs17220795	Intron	0	27/144 (18.7%)	-	
5833022	rs11094874	Intron	0	20/144 (13.8%)	-	
5833142	rs73450234	Intron	0	27/144 (18.7%)	-	
5835013	-	Intron	0.07	1/144 (0.69%)	-	
5835059	rs184731462	Intron	0	1/144 (0.69%)	-	
5835331	rs6529896	Intron	0	18/144 (12.5%)	-	
5835462	rs144552825	Intron	0	1/144 (0.69%)	-	
5835543	rs17305859	Intron	0.02	27/144 (18.7%)	-	
5835682	-	Intron	0	1/144 (0.69%)	-	
5835728	rs5916271	Intron	0	16/144 (11.1%)	-	
5836197	-	Intron	0	1/144 (0.69%)	-	
5836242	rs5915621	Intron	0.08	109/144 (75.7%)	-	
5836887	rs57586772	Intron	0	22/144 (15.2%)	-	
5837331	rs139034847	Intron	0	1/144 (0.69%)	-	
5837688	rs7052720	Intron	0	1/144 (0.69%)	-	
5837742	-	Intron	0	1/144 (0.69%)	-	
5837762	rs72627584	Intron	0	8/144 (5.5%)	-	
5952368	rs16997675	Intron	0	26/144 (18.0%)	-	
5952697	rs183334183	Intron	0.03	3/144 (2.0%)	-	
5952737	rs73184618	Intron	0.01	21/144 (14.5%)	-	
5952853	rs6529913	Intron	0	91/144 (63.2%)	-	
5952943	rs187105094	Intron	0.07	2/144 (1.3%)	-	
5954145	rs138573529	Intron	0	2/144 (1.3%)	-	
5954178	rs7052989	Intron	0	22/144 (15.2%)	-	
5954339	-	Intron	0	1/144 (0.69%)	-	
5954645	rs7057259	Intron	0	20/144 (13.8%)	-	
5956003	rs188758342	Intron	0	1/144 (0.69%)	-	
5956565	rs150691530	Intron	0.04	1/144 (0.69%)	-	
5957751	rs147450716	Intron	0	3/144 (2.0%)	-	
5958529	rs6639582	Intron	0	68/144 (47.2%)	-	
5959219	rs6638591	Intron	0	63/144 (43.7%)	-	
5959269	-	Intron	0	1/144 (0.69%)	-	
6075696	-	Intron	0.01	1/144 (0.69%)	-	
6076096	rs5915650	Intron	0	3/144 (2.0%)	-	
6076178	-	Intron	0	1/144 (0.69%)	-	
6076445	rs191813837	Intron	0	1/144 (0.69%)	-	
6076545	-	Intron	0	1/144 (0.69%)	-	
6076546	-	Intron	0	1/144 (0.69%)	-	
6077039	-	Intron	0	1/144 (0.69%)	-	
6077606	rs139601811	Intron	0	5/144 (3.4%)	-	
6078334	rs5961408	Intron	0	10/144 (6.9%)	-	
6078526	rs145089531	Intron	0	4/144 (2.7%)	-	
6079713	rs150495370	3'UTR	0	2/144 (1.3%)	-	
6079795	-	3'UTR	0	1/144 (0.69%)	-	
6079898	rs6639602	Intron	0	1/144 (0.69%)	-	
6080117	rs5961940	Intron	0.02	10/144 (6.9%)	-	
6080224	rs5961941	Intron	0	86/144 (59.7%)	-	
70279215	rs62609610	Intergenic	0	39/144 (27.0%)	-	
70279297	rs62609611	Intergenic	0	39/144 (27.0%)	-	
70279298	rs62609612	Intergenic	0	39/144 (27.0%)	-	
70279577	-	Intergenic	0.01	1/144 (0.69%)	-	
70279832	rs60090756	Intergenic	0	14/144 (9.7%)	-	
70280327	rs73634865	Intergenic	0	1/144 (0.69%)	-	
70281629	rs115732184	Intron	0.98	12/144 (8.3%)	-	
70281630	rs116209949	Intron	0.96	12/144 (8.3%)	-	
70281688	rs187697102	Intron	0.01	5/144 (3.4%)	-	
70281711	rs5981076	Intron	0.09	70/144 (48.6%)	-	
70281713	rs5981077	Intron	0.86	70/144 (48.6%)	-	
70282011	rs72628895	Intron	0	11/144 (7.6%)	-	
70282170	rs62609614	Intron	1	39/144 (27.0%)	-	
70283672	rs139269684	Intron	0	2/144 (1.3%)	-	
70283771	rs7051629	Intron	0	70/144 (48.6%)	-	
70284035	-	Intron	0	1/144 (0.69%)	-	
70284053	rs11795613	Intron	0	69/144 (47.9%)	-	
70284973	-	Intron	1	1/144 (0.69%)	1/1416 (0.07%)	
70285256	-	Intron	0.98	1/144 (0.69%)	0/1441	
70286263	rs79970703	Intron	1	6/144 (4.1%)	-	
70286468	rs147942917	Intron	0.82	2/144 (1.3%)	23/1415(1.6%)	
70286969	rs4844285	Intron	0	70/144 (48.6%)	-	
70287014	rs150925365	Intron	0	3/144 (2.0%)	-	
70287059	rs60675051	Intron	0	1/144 (0.69%)	-	
70287136	rs7051391	Intron	0	17/144 (11.8%)	-	
70287293	rs7053363	Intron	0	68/144 (47.2%)	-	
70287298	-	Intron	0	1/144 (0.69%)	-	
70288068	rs73634866	Intron	0	1/144 (0.69%)	-	
70288123	-	Intron	0	1/144 (0.69%)	-	
70288188	rs72628897	Intron	0	2/144 (1.3%)	-	
70288299	rs4844286	Intron	0	68/144 (47.2%)	-	
70288372	-	Intron	0	1/144 (0.69%)	-	
70288838	-	Intron	0.69	1/144 (0.69%)	2/1441 (0.13)	
70288980	rs5981079	Intron	0	60/144 (41.6%)	-	
70288986	rs148538905	Intron	0	3/144 (2.0%)	-	
70289941	rs2233440	Intron	1	21/144 (14.5%)	-	
70290163	rs180681274	Intron	1	2/144 (1.3%)	3/1414 (0.2%)	
70290296	rs147925556	Intron	0.86	3/144 (2.0%)	21/1417 (1.4%)	
70290861	-	Intron	0.12	1/144 (0.69%)	-	
70290929	rs7051529	Intron	0.92	65/144 (45.1%)	-	
70290947	rs12846068	Intron	0	32/144 (22.2%)	-	
70291115	-	Intron	0	1/144 (0.69%)	-	
70291342	rs186295554	Intron	0.94	6/144 (4.1%)	14/1442 (0.97%)	
70291656	-	Intron	1	1/144 (0.69%)	0/1440	
70291748	rs185402974	Intron	1	4/144 (2.7%)	26/1416 (1.8%)	
70292375	rs67640617	Intron	0	7/144 (4.8%)	-	
70299000	rs1883069	Intron	0	59/144 (40.9%)	-	
70299558	-	Intron	0.14	1/144 (0.69%)	-	
70300169	rs7886134	Intron	0	59/144 (40.9%)	-	
70300655	rs73634867	Intron	0	1/144 (0.69%)	-	
70300992	rs7051693	Intron	0	18/144 (12.5%)	-	
70301074	rs58667264	Intron	0	1/144 (0.69%)	-	
70301226	rs35106866	Intron	0	41/144 (28.4%)	-	
70301411	rs61035049	Intron	0	1/144 (0.69%)	-	
70301800	rs182467467	Intron	0.01	1/144 (0.69%)	-	
70302072	rs146439077	Intron	0	11/144 (7.6%)	-	
70302131	rs4844287	Intron	0	59/144 (40.9%)	-	
70302916	-	Intron	0.02	1/144 (0.69%)	-	
70303550	rs2233442	Intron	0	1/144 (0.69%)	-	
70304938	rs7054811	Intron	0	2/144 (1.3%)	-	
70305280	rs73539640	Intron	0	5/144 (3.4%)	-	
70305367	rs7881425	Intron	0.02	18/144 (12.5%)	-	
70305373	rs7879983,
rs67502493	Intron	0.01	18/144 (12.5%)	-	
70305637	rs146987174	Intron	0	2/144 (1.3%)	-	
70305701	-	Intron	0	1/144 (0.69%)	-	
70306734	-	3'UTR	0.177	1/144 (0.69%)	-	
70306764	-	3'UTR	0.42	1/144 (0.69%)	-	
70306767	-	3'UTR	0.14	1/144 (0.69%)	-	
70306922	-	3'UTR	0.73	1/144 (0.69%)	0/1440	
70308327	-	Intergenic	0.19	1/144 (0.69%)	-	
