# Supplementary material for: Identification of rare X-linked neuroligin variants by massively parallel sequencing in males with autism spectrum disorder
Source: Mol Autism. 2012 Sep 28;3:8. doi: 10.1186/2040-2392-3-8 (PMC3492087; doi:10.1186/2040-2392-3-8)
Supplement: Additional file 3 — Table showing small insertion and deletion variants detected in 144 males with diagnosis of autism from the Autism Genetic Resource Exchange. Contains variant position, dbSNP ID, functional annotation, and frequency in cases and controls. [file 2040-2392-3-8-S3.rtf]

Supplementary Table 3. Small insertions and deletions (indels) detected in 144 males with a diagnosis of autism from the Autism Genetic Resource Exchange (AGRE).

Variant Start Position (hg18)	Variant Stop Position (hg18)	dbSNP ID (dbSNP 135)	Functional Class	Patient Frequency	
5820149	5820150	-	UTR  	1/144(0.69%)	
5816038	5816042	-	Intergenic 	1/144(0.69%)	
5816163	5816164	-	Intergenic 	23/144(0.16%)	
5816694	5816695	-	Intergenic 	23/144(0.16%)	
5816727	5816732	 rs57512078	Intergenic 	63/144(0.44%)	
5817175	5817176	-	Intergenic 	23/144(0.16%)	
5822796	5822796	-	Intronic  	1/144(0.69%)	
5825962	5825963	rs144612614	Intronic  	64/144(0.44%)	
5826078	5826081	-	Intronic  	3/144(0.02%)	
5826164	5826167	rs142504588	Intronic  	112/144(0.78%)	
5833370	5833374	-	Intronic  	1/144(0.69%)	
5834078	5834080	rs72214951	Intronic  	2/144(0.01%)	
5835681	5835681	-	Intronic  	1/144(0.69%)	
5837765	5837765	rs71853983	Intronic  	20/144(0.14%)	
5954284	5954284	-	Intronic  	1/144(0.69%)	
5955475	5955479	-	Intronic  	1/144(0.69%)	
5955694	5955694	rs34369385	Intronic  	7/144(0.05%)	
5958435	5958435	rs67640959	Intronic  	68/144(0.47%)	
5959006	5959006	-	Intronic  	1/144(0.69%)	
5959410	5959410	-	Intronic  	1/144(0.69%)	
6076092	6076092	-	Intronic  	1/144(0.69%)	
6076130	6076134	-	Intronic  	1/144(0.69%)	
6078518	6078525	-	Intronic  	2/144(0.01%)	
70279633	70279635	-	Intergenic 	1/144(0.69%)	
70287122	70287122	-	Intronic  	1/144(0.69%)	
70298626	70298627	-	Intronic  	5/144(0.03%)	
70303034	70303034	-	Intronic  	1/144(0.69%)	
70305323	70305326	-	Intronic  	1/144(0.69%)	
70305390	70305390	-	Intronic  	20/144(0.14%)	
70305433	70305457	rs71889719	Intronic  	19/144(0.13%)	
70305458	70305458	rs59138595	Intronic  	18/144(0.13%)	
70305472	70305473	rs66484118	Intronic  	1/144(0.69%)	
