# Supplementary material for: Identification of rare X-linked neuroligin variants by massively parallel sequencing in males with autism spectrum disorder
Source: Mol Autism. 2012 Sep 28;3:8. doi: 10.1186/2040-2392-3-8 (PMC3492087; doi:10.1186/2040-2392-3-8)
Supplement: Additional file 4 — Table showing NLGN3 and NLGN4X 3’UTR genetic variation from the 1000 Genomes database. Contains variant position, dbSNP ID, PhastCons score, and estimated frequency. [file 2040-2392-3-8-S4.rtf]

Supplementary Table 4. NLGN3 and NLGN4X 3'UTR single nucleotide variants (SNVs) and insertion-deletions (Indels) from the 1000 Genomes database.


Type	Position	SNV ID	PhastCons	Estimated Frequency	
SNV	X:5808082	rs182287430	1	0.001	
SNV	X:5808355	rs186894475	1	0.001	
SNV	X:70390226	rs6624536	1	ND	
SNV	X:70390986	rs2880110	1	ND	
SNV	X:70389951	1000GENOMES_X_70389951	0.957	ND	
SNV	X:5808456	rs3723	0.886	ND	
SNV	X:70390903	rs2472192	0.87	ND	
SNV	X:70390334	rs185842785	0.61	0.001	
SNV	X:70390907	rs78195299	0.563	0.005	
SNV	X:70390613	rs180705212	0.528	0.001	
SNV	X:5810853	rs4995614	0.039	ND	
SNV	X:5810090	rs3810685	0.035	0.39	
SNV	X:5810121	rs184042624	0.028	0.002	
SNV	X:70390589	rs190402896	0.024	ND	
SNV	X:70390183	rs193068757	0.012	0.004	
SNV	X:5810346	rs190728907	0.008	ND	
SNV	X:5810684	rs3810687	0.008	0.112	
SNV	X:5808811	rs189038197	0.004	0.001	
SNV	X:5810342	rs188582063	0.004	0.001	
SNV	X:5808861	rs1882260	0	0.275	
SNV	X:5808871	rs16983882	0	0.174	
SNV	X:5808913	rs73450215	0	0.016	
SNV	X:5809012	rs185528611	0	0.002	
SNV	X:5809016	rs189555334	0	0.001	
SNV	X:5809156	rs114253047	0	0.004	
SNV	X:5809384	rs41309587	0	0.005	
SNV	X:5809396	rs5961883	0	0.034	
SNV	X:5809574	rs41305199	0	0.002	
SNV	X:5809620	rs186141923	0	0.002	
SNV	X:5809956	rs192258189	0	ND	
SNV	X:5810299	rs5916269	0	0.153	
SNV	X:5810401	rs112340691	0	ND	
SNV	X:5810574	rs3810686	0	0.383	
SNV	X:5810735	rs182561696	0	0.002	
SNV	X:5810756	rs3810688	0	0.344	
SNV	X:5810841	rs4995615	0	ND	
SNV	X:70389969	ESP_X_70389969	0	0	
SNV	X:70390639	rs183671009	0	0.001	
INDEL	X:5808544	rs66739111	0	ND	
INDEL	X:5808698	rs67871710	0	ND	
INDEL	X:5808854	rs67783080	0	ND	
INDEL	X:5809653	rs66496124	0.343	ND	
INDEL	X:5809732	rs67871435	0	ND	
INDEL	X:5809737	rs68187559	0	ND	
INDEL	X:5808302 -5808303	1000GENOMES_X_5808301	0/0.004	ND	
INDEL	X:70390029 -70390028	rs35156036	0	ND	
INDEL	X:70390019 -70390018	rs68176212	0.004/0.004	ND	
INDEL	X:70390008 -70390007	rs112095862	0.039/0	ND	
INDEL	X:70390007 -70390006	1000GENOMES_X_70390006	0/0.039	ND	
