# Supplementary material for: Identification of rare X-linked neuroligin variants by massively parallel sequencing in males with autism spectrum disorder
Source: Mol Autism. 2012 Sep 28;3:8. doi: 10.1186/2040-2392-3-8 (PMC3492087; doi:10.1186/2040-2392-3-8)
Supplement: Additional file 5 — Figure showing segregation analysis of the highly conserved NLGN3 and NLGN4X UTR variants. The segregation of the highly conserved UTR variants in NLGN3 (chrX:70306922) and NLGN4X (chrX:5818136, chrX:5820149–50) with a diagnosis of autism were checked by sequencing the mother and affected and unaffected male siblings of the corresponding proband. The top base shown is the reference base, the bottom base shown is the variant base. Both the NLGN4X variants segregated with autism. We also tested segregation of the two control variants (chrX:70306764, chrX:70306767) with autism. DNA was not available for the unaffected sibling of the proband carrying the NLGN4X UTR variant (chrX:5820149–150). [file 2040-2392-3-8-S5.pdf]

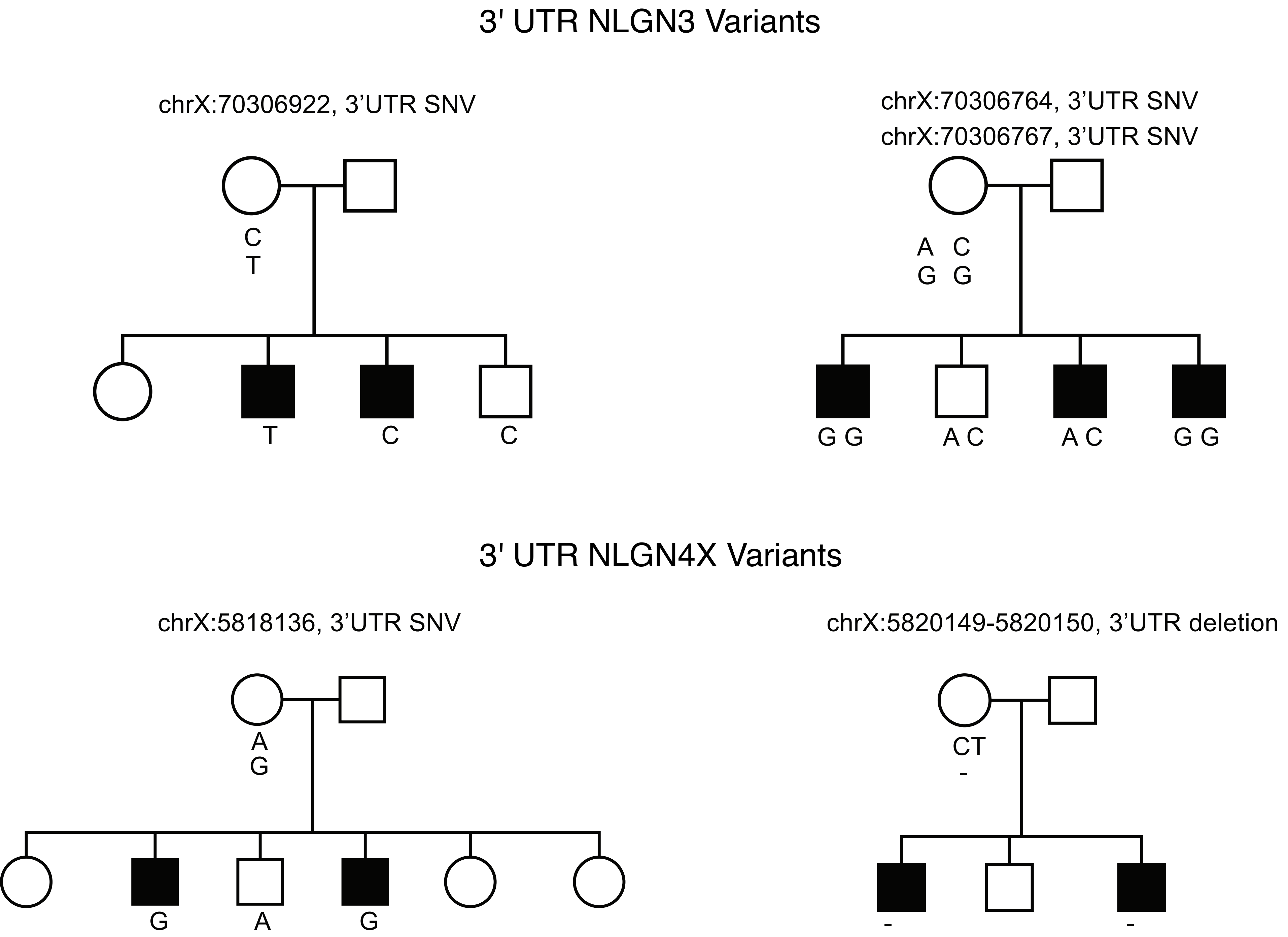
3' UTR NLGN3 Variants
chrX:70306922, 3'UTR SNV
C
T
T
C
C
chrX:70306764, 3'UTR SNV
chrX:70306767, 3'UTR SNV
A C
G G
G G
A C
A C
G G
3' UTR NLGN4X Variants
chrX:5818136, 3'UTR SNV
A
G
G
A
G
chrX:5820149-5820150, 3'UTR deletion
CT
-
-
-
