# Supplementary figures and images for: Identification of rare X-linked neuroligin variants by massively parallel sequencing in males with autism spectrum disorder
Source: Mol Autism. 2012 Sep 28;3:8. doi: 10.1186/2040-2392-3-8 (PMC3492087; doi:10.1186/2040-2392-3-8)

## NLGN3 Intronic Variant Tested

chrX:70291656, Intronic SNV

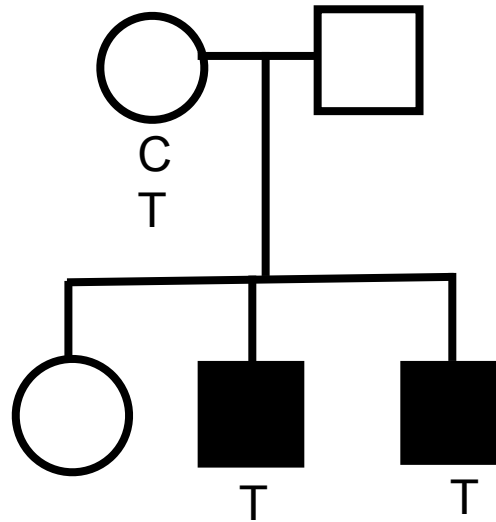

Supplement: Additional file 6 — Figure showing segregation analysis of two highly conserved NLGN3 intronic variants. The segregation of NLGN3 intronic variants (chrX:70291656) and (chrX:70284973) with a diagnosis of autism. Both are located within transcription factor binding sites (TFBS). [file 2040-2392-3-8-S6.pdf]

A

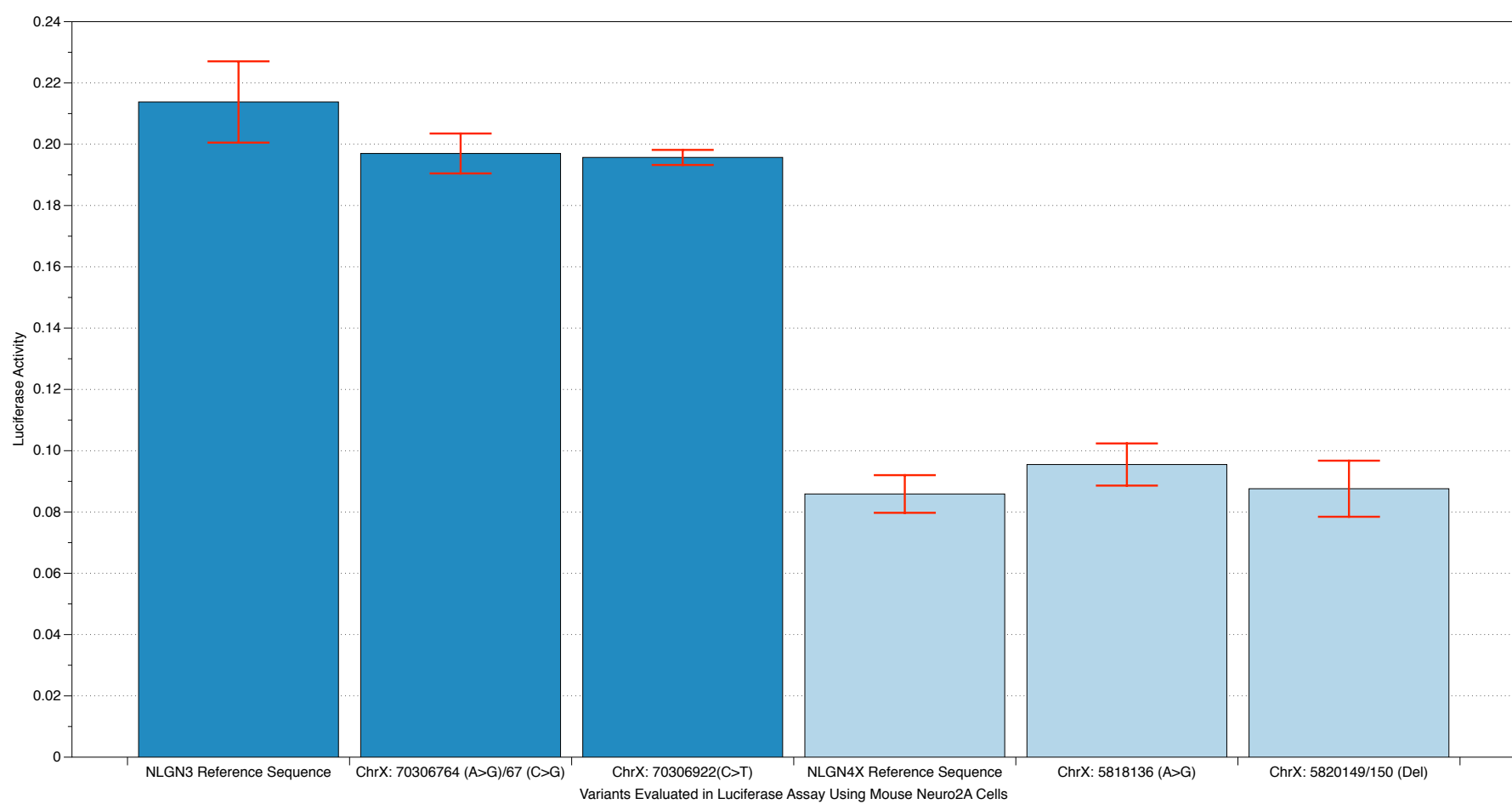

B

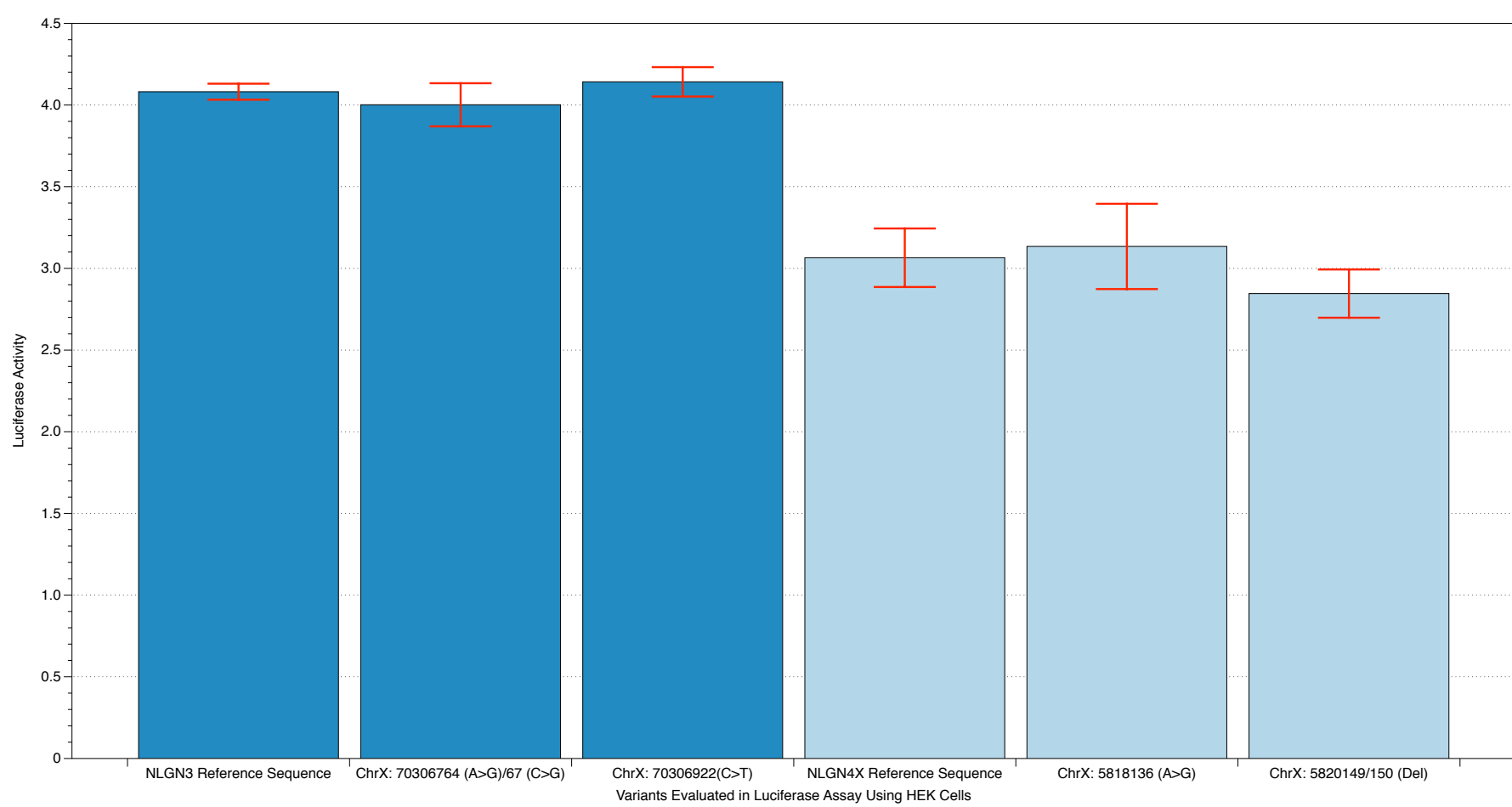

Supplement: Additional file 8 — Figure showing the results of luciferase assays for one 3’UTR variant in NLGN3 and two 3’UTR variants in NLGN4X. (A). Results of luciferase expression assay in Neuro2a cell lines. These constructs were tested against the reference allele constructs using a luciferase reporter assay. One of the NLGN3 constructs (chrX:70306764/67) serves as a control for the variant at a non-conserved site. Error bars show 2 standard errors on either side of the mean value. (B). Results of luciferase expression assay in human embryonic kidney (HEK) cell lines. The experiment was similar to that described above. [file 2040-2392-3-8-S8.pdf]
