# Supplementary material for: Identification of rare X-linked neuroligin variants by massively parallel sequencing in males with autism spectrum disorder
Source: Mol Autism. 2012 Sep 28;3:8. doi: 10.1186/2040-2392-3-8 (PMC3492087; doi:10.1186/2040-2392-3-8)
Supplement: Additional file 7 — Table showing sequenom genotyping assay primers for single nucleotide variants discovered in NLGN3 and NLGN4X loci. This table contains detailed information about each primer pair and extension primer. [file 2040-2392-3-8-S7.rtf]

Supplementary Table 4. Sequenom genotyping assay primers for single nucleotide variants (SNVs) discovered at the NLGN3 and NLGN4X loci.

Variant	Amplification Primer1	Amplification Primer 2	Extension Primer	
NLGN3_70306922 C>T	ACGTTGGATGTGCTGTTTCATGGGTCCTCG	ACGTTGGATGATACGGAAGTAAACCTGAAC	ACGCAGATGAGTCCT	
NLGN4X_5818136 A>G	ACGTTGGATGTAAAAATGCTGCTGAAAAG	ACGTTGGATGTTGCCTTAAGTAATGATAG	ggATGGCCGGACACATA	
 NLGN3_70284973 T>G	ACGTTGGATGTCTTCCCTCTCCCAGCATTG	ACGTTGGATGCCCTCCTGGCAACAGTGTC	GCAACAGTGTCACTCAC	
NLGN3_70285256 G>A	ACGTTGGATGTCCCACCATCTCCTTGTTTC	ACGTTGGATGCTCTTTCACTAGCTGATGCC	ccGATGCCTCCTCCCGC	
NLGN3_70286468 C>T	ACGTTGGATGAGAGGGTTGTAGGGATCAAG	ACGTTGGATGGGGAGAATTGGAACAGTAAC	CAGTAACAGCCTAAGGAA	
NLGN3_70288838 C>G	ACGTTGGATGATCGCCAGGATGCACTTCTA	ACGTTGGATGGGAGACATTTAGGATCTGGG	ttCACAGATTCTCCTTCCT	
NLGN3_70290163 C>T	ACGTTGGATGAACACACCAACGGACAGACA	ACGTTGGATGGCTGGTGCATGGCACAGAG	GGCACAGAGCCCCTC	
NLGN3_70290296 A>G	ACGTTGGATGATCTGTCTTTGGCCCTGTTG	ACGTTGGATGTCTGCTTGTGGTCCCCTGCT	TGCTCCTGCCAGCCA	
NLGN3_70291342 G>A	ACGTTGGATGTCTTGTCTCTGTCTGCACTG	ACGTTGGATGCCCAAGACTAGACCAGTTAC	TAGACCAGTTACATGGAAAAA	
NLGN3_70291656 C>T	ACGTTGGATGCAGCAAAGCTATCCCAGGTG	ACGTTGGATGAGCTCCAGGTTGAGCAACCC	gggatGTTGAGCAACCCCATGAGT	
NLGN3_70291748 G>A	ACGTTGGATGATAGCTTTGCTGCCCGCAC	ACGTTGGATGGTGAGACACAGGCTGGGT	GGCTGGGTGGGATGC	
